# Hyperbaric oxygen treatment in autism spectrum disorders

**DOI:** 10.1186/2045-9912-2-16

**Published:** 2012-06-15

**Authors:** Daniel A Rossignol, James J Bradstreet, Kyle Van Dyke, Cindy Schneider, Stuart H Freedenfeld, Nancy O’Hara, Stephanie Cave, Julie A Buckley, Elizabeth A Mumper, Richard E Frye

**Affiliations:** 1 Rossignol Medical Center, 3800 West Eau Gallie Blvd., Melbourne, FL, 32934, USA; 2 International Child Development Resource Center, 104 Colony Park Dr. Suite 600, Cumming, GA, 30040, USA; 3 Southwest College of Naturopathic Medicine, Department of Pediatrics, Tempe, AZ, USA; 4 Wisconsin Integrative Hyperbaric Center, 6200 Nesbitt Road, Fitchburg, WI, 53719, USA; 5 Center for Autism Research and Education, 4045 East Union Hills Drive, Suite 116, Phoenix, AZ, 85050, USA; 6 Stockton Family Practice, Stockton Center for Health Care, 56 South Main Street, Suites A & B, Stockton, NJ, 08559, USA; 7 Center for Autism & Integrative Health, 3 Hollyhock Lane, Wilton, CT, 06897, USA; 8 Cypress Integrative Medicine, 10562 South Glenstone Place, Baton Rouge, LA, 70810, USA; 9 Pediatric Partners of Ponte Vedra, 5270 Palm Valley Road, Ponte Vedra Beach, FL, 32082, USA; 10 The Rimland Center, 2919 Confederate Ave, Lynchburg, VA, 24501, USA; 11 Department of Pediatrics, Arkansas Children’s Hospital Research Institute, University of Arkansas for Medical Sciences, Little Rock, AR, 72202, USA

**Keywords:** Hyperbaric oxygen treatment, Autism, Oxidative stress, Inflammation, Behavior

## Abstract

Traditionally, hyperbaric oxygen treatment (HBOT) is indicated in several clinical disorders include decompression sickness, healing of problem wounds and arterial gas embolism. However, some investigators have used HBOT to treat individuals with autism spectrum disorders (ASD). A number of individuals with ASD possess certain physiological abnormalities that HBOT might ameliorate, including cerebral hypoperfusion, inflammation, mitochondrial dysfunction and oxidative stress. Studies of children with ASD have found positive changes in physiology and/or behavior from HBOT. For example, several studies have reported that HBOT improved cerebral perfusion, decreased markers of inflammation and did not worsen oxidative stress markers in children with ASD. Most studies of HBOT in children with ASD examined changes in behaviors and reported improvements in several behavioral domains although many of these studies were not controlled. Although the two trials employing a control group reported conflicting results, a recent systematic review noted several important distinctions between these trials. In the reviewed studies, HBOT had minimal adverse effects and was well tolerated. Studies which used a higher frequency of HBOT sessions (e.g., 10 sessions per week as opposed to 5 sessions per week) generally reported more significant improvements. Many of the studies had limitations which may have contributed to inconsistent findings across studies, including the use of many different standardized and non-standardized instruments, making it difficult to directly compare the results of studies or to know if there are specific areas of behavior in which HBOT is most effective. The variability in results between studies could also have been due to certain subgroups of children with ASD responding differently to HBOT. Most of the reviewed studies relied on changes in behavioral measurements, which may lag behind physiological changes. Additional studies enrolling children with ASD who have certain physiological abnormalities (such as inflammation, cerebral hypoperfusion, and mitochondrial dysfunction) and which measure changes in these physiological parameters would be helpful in further defining the effects of HBOT in ASD.

## Introduction

Hyperbaric oxygen treatment (HBOT) involves inhaling up to 100% oxygen at a pressure greater than one atmosphere (atm) in a pressurized chamber [1]. HBOT is indicated in several clinical disorders include decompression sickness, healing of problem wounds, arterial gas embolism and carbon monoxide poisoning [2]. Treatment with HBOT for these disorders uses higher pressures (over 2.0 atm). Higher pressure HBOT has been shown to increase the oxygen content of plasma [3] and body tissues [4] and may normalize oxygen levels in ischemic tissues [5].

As compared to treatment with HBOT for many classical indications, HBOT used at lower pressures (e.g. 1.3 to 1.5 atm and oxygen at 24 to 100%) has started to be investigated to treat certain neurological disorders, some of which are considered to have few efficacious treatments. For example, recent studies have investigated lower pressure HBOT for traumatic brain injury (TBI) in both animal models [6-10] and humans [11-23]. In a recent prospective trial of 16 patients with TBI, HBOT at 1.5 atm/100% oxygen (40 hourly treatments over 30 days) resulted in significant improvements in their neurological exam, IQ, memory, post-traumatic stress symptoms, depression, anxiety and quality of life. Patients also displayed objective improvements in brain perfusion measured by pre- and post-HBOT single photon emission computed tomography (SPECT) scans [17]. The human studies of TBI also include a controlled retrospective review and a controlled prospective clinical trial [15,18]. Larger multicenter trials are ongoing in attempt to confirm these controlled clinical studies [24].

Other neurological disorders have been reported to improve with lower pressure HBOT; one investigator reported significant improvements in IQ for a 15 year old child who had fetal alcohol syndrome using HBOT at 1.5 atm/100% oxygen for 73 sessions [25]. Some investigators have reported that HBOT possesses neuroprotective effects [8,26,27]. Interestingly, oxygen supplementation has recently been reported to enhance cognition [17]. For example, in several double-blind studies of healthy young adults, the use of supplementary oxygen, when compared with room air, significantly enhanced memory [28], cognitive performance, word recall and reaction time for 24 hours [29], as well as attention and picture recognition [30].

Autism spectrum disorders (ASD) are a heterogeneous group of neurodevelopmental disorders that are defined by behavioral observations and are characterized by impairments in communication and social interaction along with restrictive and repetitive behaviors [31]. ASD includes autistic disorder, Asperger syndrome, and pervasive developmental disorder-not otherwise specified (PDD-NOS). An estimated 1 out of 110 individuals in the United States is currently affected with an ASD [32]. The etiology of ASD is unclear at this time. Although several genetic syndromes, such as Fragile X and Rett syndromes, have been associated with ASD, empirical studies have estimated that genetic syndromes only account for 6-15% of ASD cases [33]. Therefore, the majority of ASD cases are not due to a simple single gene or chromosomal disorder. Although many of the cognitive and behavioral features of ASD are thought to arise from dysfunction of the central nervous system (CNS), evidence from many fields of medicine has documented multiple non-CNS physiological abnormalities associated with ASD [34-37], suggesting that, in some individuals, ASD arises from systemic, rather than organ specific, abnormalities. Specifically, in recent decades, research and clinical studies in ASD have implicated physiological and metabolic systems that transcend specific organ dysfunction, such as cerebral hypoperfusion, immune dysregulation, inflammation, oxidative stress, and mitochondrial dysfunction [38,39]. In this context, ASD may arise from, or at least involve, systemic physiological abnormalities rather than being a purely CNS disorder, at least in a subset of individuals with ASD [40].

To date, ASD has few efficacious treatments. Applied Behavioral Analysis (ABA) is a form of behavioral therapy which has been reported to lead to improvements in some children with ASD. ABA treats the behavioral manifestations of ASD. Studies of ABA generally observe children with ASD over a period of one to two years. Lovaas first reported that ABA resulted in significant gains in IQ and behavioral problems over a 2 year period of time in some children with ASD [41]. Researchers from the Wisconsin Early Autism Project observed similar behavioral and IQ improvements with ABA after 4 years of observations [42]. However, only modest gains were detected using ABA compared to eclectic therapy by researchers in Norway when children were observed for only one year [43]. Therefore, behavioral therapies typically require long time periods to cause behavioral and cognitive changes in children with ASD.

Some treatments for ASD target physiological abnormalities that have been reported in some children. However, very few of these types of treatments have been critically evaluated. Starting around 2005, some investigators speculated that HBOT may be useful in improving behavioral and physiological abnormalities found in some children with ASD [44-48]. This manuscript will review the current evidence for HBOT as a treatment for ASD. First, the effects of HBOT on physiological abnormalities in children with ASD will be reviewed. Secondly, the effects on autistic behaviors will be discussed. Finally, potential adverse effects of HBOT in ASD and limitations of studies will be reviewed.

### The effects of HBOT on physiological abnormalities in ASD

#### Cerebral hypoperfusion in ASD and the effects of HBOT

A number of studies have reported cerebral hypoperfusion in individuals with ASD compared to controls as measured by positron emission tomography (PET), SPECT or functional magnetic resonance imaging (fMRI) [49-51]. This hypoperfusion has been correlated with certain autistic behaviors such as repetitive behaviors [52], desire for sameness [53], impairments in processing facial expressions and emotions [54], and decreased language development [55]. Furthermore, lower cerebral perfusion has been significantly correlated with increasing age in children with ASD [55] and with more severe autistic behaviors [56].

**Figure 1:**
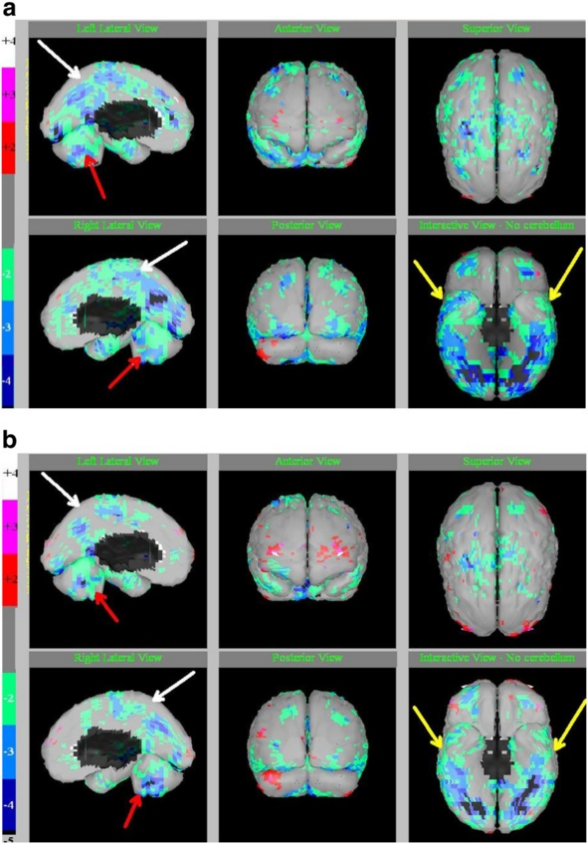
**SPECT scan images in a 12 year old boy with autism (a) before and (b) after 80 sessions of HBOT at 1.3 atm.** Legend: minus 2 (green) to minus 4 (blue) standard deviations indicate the magnitude of regional hypofunctioning (hypoperfusion). White arrows indicate improvement in deeper cortical hypoperfusion patterns. Red arrows on sagittal slices show the midline cerebellum hypoperfusion and improvements after HBOT. Yellow arrows on the “underside” view show the temporal lobe hypoperfusion with improvements after HBOT. Pictures courtesy of J. Michael Uszler, MD. Credit: Permission for use of figure from Hyperbaric Oxygen for Neurological Disorders granted by Best Publishing Company, Palm Beach Gardens, FL.

It is possible that HBOT could improve cerebral perfusion in ASD. Several studies have reported significant improvements in cerebral perfusion with the use of HBOT at lower pressures (i.e., 1.3 to 1.5 atm), as measured by pre- and post-HBOT SPECT scans in several neurological conditions, including TBI and chronic brain injury [16,17,19,22]. In addition, cerebral perfusion has been shown to change in children with ASD after treatment with HBOT. For example, several case reports have demonstrated improvements in cerebral perfusion, as measured by pre- and post-treatment SPECT scans, with the use of HBOT at 1.3 atm/24% oxygen, including one child with ASD who received 1 hour of HBOT per day for 10 consecutive days [57] and two children with ASD who received 40–80 treatments [58]. Behavioral improvements were observed in these children as well.Figure 1a-b demonstrates the pre- and post-HBOT SPECT scans in one child from this latter case report [58].

Kinaci et al. reported on the cerebral perfusion effects of HBOT at 1.5 atm/100% oxygen for 50 sessions at 60 minutes per day in 108 children with ASD [59]. At baseline, all 108 patients had normal MRI scans and decreased temporal lobe perfusion as measured by SPECT scans, while 88% had decreased frontal lobe perfusion and 61% had decreased perfusion to other brain areas. Post-treatment HBOT SPECT scans demonstrated that 82.4% of the patients had an improvement in temporal lobe perfusion, 85.3% improved in frontal lobe perfusion, and 75.8% had improvements in perfusion to other brain areas. Behavioral improvements were also observed in these children. Strengths of this study included objective measurements (SPECT imaging), evaluations by clinicians, and a larger sample size than other studies.

#### Inflammation in ASD and the effects of HBOT

Recent studies support the notion that some individuals with ASD manifest neuroinflammation, immune dysregulation and/or gastrointestinal inflammation. A recent review reported that 416 publications implicated inflammation or immune abnormalities in ASD, including 65 publications of neuroinflammation and 31 publications of gastrointestinal inflammation [38]. A number of studies suggest that the gastrointestinal inflammation reported in some children with ASD has characteristics similar to inflammatory bowel disease (IBD) [60-63]. Furthermore, several studies have reported abnormal inflammatory markers in some children with ASD. For example, elevations in TNF-alpha [64-67] and neopterin (a marker of activation of the cellular immune system) [68-70] have been reported in several studies of children with ASD.

Treatment with HBOT has been shown to possess potent anti-inflammatory properties in both animal [71-73] and human studies [74-78]. HBOT has been reported to decrease the production of pro-inflammatory cytokines (including TNF-alpha, interferon-gamma, IL-1 and IL-6) in both animal [79,80] and human studies [78,81] as well as increase counter-inflammatory IL-10 levels [82]. In one study, HBOT also decreased neopterin levels [83]. Furthermore, a recent systematic review reported improvements in studies that used HBOT in IBD [84]. The effect of HBOT on reducing inflammation may be mediated through a pressure-related effect and not necessarily by the oxygen delivered. For example, one human study reported a reduction in interferon-gamma production by lymphocytes with HBOT at 2.0 atm/10.5% oxygen but an increase in interferon-gamma with 100% oxygen delivered at 1.0 atm [78].

Two prospective studies have examined the effects of HBOT on biomarkers of inflammation in children with ASD [85,86]. In the first study, 12 children received HBOT at 1.3 atm/24% oxygen and 6 children received HBOT at 1.5 atm/100% oxygen. Biomarkers were measured before and after 40 HBOT sessions [85]. C-reactive protein (a general marker of inflammation) dropped in the overall study population (p = 0.021). Children who had the highest C-reactive protein levels showed the largest decrease. Behavioral improvements were also observed in these children.

In the second study, plasma cytokine levels, including some associated with inflammation, were measured before and after 80 HBOT sessions delivered at 1.5 atm/100% oxygen over a 20 week period in 10 children with ASD [86]. Behavioral improvements were observed in these children, but the study reported no significant changes in cytokines during the study. However, the authors noted that none of the children had abnormal cytokine levels at the beginning of the study, making it less likely that a significant change could be observed. Furthermore, since cerebrospinal fluid (CSF) cytokine abnormalities have been reported in some children with ASD [64,87,88], the authors noted that CSF cytokines could have changed. However, CSF cytokines were not measured in the study. Further studies of HBOT in children with ASD who have abnormal cytokines and markers of inflammation are warranted to investigate these findings in more depth.

In addition to these two studies, one of the authors (DAR) has observed a decrease in urinary neopterin levels after HBOT in some children. For example, one child with ASD who was treated with HBOT at 1.5 atm/100% oxygen for 40 treatments over 1 month had a drop in urinary neopterin, measured immediately before starting HBOT and immediately after stopping, from 768 to 391 μmol/mol creatinine, respectively. Another child with ASD who had significant eczema and bowel inflammation with abdominal distension had resolution of eczema, chronic diarrhea and abdominal distension with HBOT at 1.5 atm/100% oxygen for 40 treatments over 1 month [58]. SeeFigure 2a-b for pre- and post-HBOT pictures of this child.

**Figure 2:**
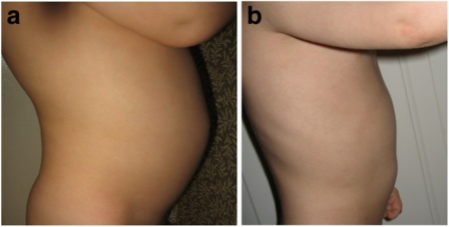
**6 year old boy with autism who received HBOT at 1.5 atm. Before HBOT, physical exam reveals distended abdomen (a) with chronic diarrhea.** After HBOT, patient has improvements in distended abdomen (**b**) and bowel movements. Figure use with parental permission. Credit: Permission for use of figure from Hyperbaric Oxygen for Neurological Disorders granted by Best Publishing Company, Palm Beach Gardens, FL.

#### Mitochondrial dysfunction in ASD and the effects of HBOT

Some individuals with ASD manifest evidence of mitochondrial dysfunction [34,89]. A recent review article reported that 145 publications implicated mitochondrial dysfunction in ASD [38]. Although treatments for mitochondrial dysfunction remain relatively limited [34], interest has recently increased in using HBOT as a potential treatment. Both animal and human studies have examined the effects of HBOT on mitochondrial function. Several animal models have reported improvements in mitochondrial function with HBOT [90-96]. For example, in one study of rats with normal mitochondrial function, HBOT increased the production of ATP in muscle tissue compared to a control group [97]. A recent study of rat hippocampus reported that HBOT increased mitochondrial biogenesis and autophagy through, in part, an increased production of reactive oxygen species (ROS). Through this process, new healthy mitochondrial were produced and old dysfunctional mitochondrial were destroyed. This study also found increased activation of mitochondrial DNA transcription and replication with HBOT [98]. In a recent controlled study of 69 patients with severe TBI, HBOT at 1.5 atm/100% oxygen significantly increased brain oxygen levels, increased cerebral blood flow, and decreased CSF lactate levels (high CSF lactate is a marker of mitochondrial dysfunction). In this study, HBOT also improved brain metabolism and mitochondrial function compared with both room air treatment and 100% oxygen given at normobaric pressure [18]. Although one investigator has reported improvements using HBOT in children with concomitant mitochondrial disease and ASD [99], no clinical studies have been published to date examining the effects of HBOT on mitochondrial function in individuals with ASD; further study in this area is needed.

#### Oxidative stress in ASD and the effects of HBOT

Multiple studies have reported evidence of oxidative stress in children with ASD [36,100-102]. A recent review article reported that 115 publications implicated oxidative stress in ASD [38]. Since some children with ASD have evidence of elevated oxidative stress [38,100], some investigators have expressed concern that HBOT could increase oxidative stress in this subset of children [85]. Theoretically, HBOT might increase oxidative stress through the augmented production of ROS from the high concentration of oxygen [103]. This may occur because increased oxygen delivery to mitochondria can increase ROS production. However, HBOT has been shown to upregulate the production of antioxidant enzymes such as superoxide dismutase [104,105], glutathione peroxidase [106], catalase [107], paraoxonase [108] and heme-oxygenase 1 [109,110]. This increase in antioxidant enzyme levels has been termed “conditioning” and can protect against damage caused by ROS [44,111]. Interestingly, increasing ROS may be a potential mechanism of action of HBOT because ROS play an important role in cellular signaling and in triggering certain metabolic pathways [112]. Furthermore, as previously discussed, a slight increase in ROS produced by HBOT may be beneficial as these ROS appear to augment mitochondrial biogenesis [98].

Two studies have reported measurements of oxidative stress markers before and after HBOT in children with ASD [85,113]. In the first study, HBOT was administered daily at 1.3 atm to 48 children with ASD, and superoxide dismutase (SOD), catalase and glutathione peroxidase levels were measured before starting HBOT and after 1 day and 32 days of HBOT [113]. SOD was 4.5-fold and 4.7-fold higher at 1 and 32 days after starting HBOT, respectively. Mean catalase increased by 1.9-fold after 1 day and after 32 days was 90% of the initial level before beginning HBOT. Finally, mean glutathione peroxidase increased by 1.4-fold after 1 day and after 32 days was 1.2-fold higher than before beginning HBOT. The effects of HBOT on these antioxidant enzymes may be an example of conditioning as previously discussed.

In the second prospective study, 12 children with ASD received HBOT at 1.3 atm/24% oxygen and 6 children received HBOT at 1.5 atm/100% oxygen. Biomarkers were measured before and after 40 HBOT sessions [85]. Behavioral improvements were observed in these children and plasma oxidized glutathione levels did not significantly change at 1.3 atm (p = 0.557) or 1.5 atm (p = 0.583). Since oxidized glutathione is exported from cells when intracellular levels exceed the redox capacity [114], this finding suggests that intracellular oxidative stress did not significantly worsen with HBOT at these two commonly used lower HBOT pressures in ASD [85].

### The effects of HBOT on behavioral measurements in ASD

The majority of treatment studies using HBOT in children with ASD have measured behavioral rather than physiological parameters. These behavioral studies can be divided into those with and without control children.

#### *Studies lacking control children*

Several case studies have reported behavioral improvements in individuals with ASD from treatment with HBOT. The first published report of the use of HBOT in an individual with ASD was in 1994 [115]. In this report, treatment with HBOT resulted in improvements in mood and social interactions in a three year old child with ASD. The number of treatments and other HBOT parameters were not reported. In 2002, Heuser et al. reported a “striking improvement” in behavior, memory, social interaction, verbalizations and cognitive functioning in a 4 year old boy with ASD after HBOT treatment at 1.3 atm/24% oxygen for 10 consecutive days [57]. Another investigator observed significant objective improvements in coloring skills (seeFigure 3a-d) as well as speech and self-help skills in a 17 year old child with ASD using HBOT at 1.5 atm/100% oxygen for 20 sessions [116]. Burke noted improvements in 2 children with ASD using HBOT at 1.3 atm/28% oxygen, including improvements in communication, aggressiveness and social interaction [117]. Another report noted objective improvements in one child with ASD in handwriting (Figure 4a-b) after 40 treatments with HBOT at 1.3 atm/24% oxygen, as well as improvements in fine motor skills, bowel function, language and communication [58]. One investigator reported improvements in language, social interaction and overall cognition in a 3 year old boy with ASD using HBOT at 1.3 atm/24% oxygen for 40 treatments. This child also had chronic diarrhea and had the first normal bowel movement in his life with HBOT treatment [99]. In another report, 23 patients with ASD had various improvements in social interaction, language and repetitive behaviors with HBOT at 1.5 atm [47]. Finally, one prospective study of 20 children with ASD reported improvements in communication, social interaction and stereotypical behaviors after 20 HBOT sessions at 1.5 atm/100% oxygen [118].

**Figure 3:**
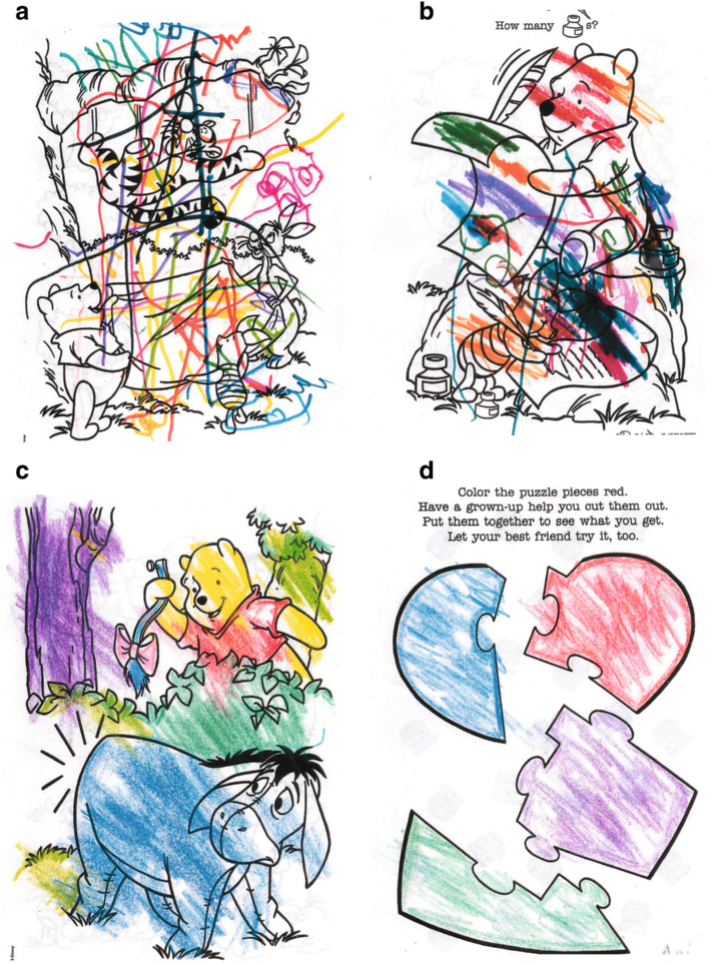
**Coloring book pages from 17 year old girl with autism: (a) before beginning HBOT at 1.5 atm/100% oxygen; (b) after one week of HBOT (5 sessions at one hour each), she is beginning to create patches of color to fill in a space; (c) after 3 weeks of HBOT (about 15 hours of HBOT), she uses correct colors for Winnie the Pooh and Eyore, and the foliage except for the tree trunk; and (d) after 5 weeks of HBOT (20 hours of HBOT), she begins to respect borders and boundaries and even outlines the inner border with color.** After 6 months, her coloring abilities remained stable. Pictures courtesy of Carol L. Henricks, MD. Credit: Permission for use of drawings granted by the Journal of American Physicians and Surgeons.

**Figure 4:**
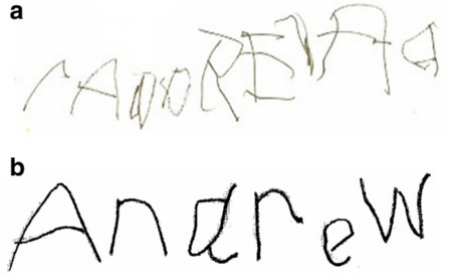
**Handwriting in a 6 year old boy (a) before and (b) after 40 HBOT sessions at 1.3 atm**. Pictures courtesy of James Neubrander, MD. Credit: Permission for use of figure from Hyperbaric Oxygen for Neurological Disorders granted by Best Publishing Company, Palm Beach Gardens, FL.

The first published case-series to examine the effects of HBOT in 6 children with ASD administered HBOT at 1.3 atm/28% oxygen (1 hour treatments for 40 treatments) [119]. Improvements were reported on the Autism Treatment Evaluation Checklist (ATEC), the Childhood Autism Rating Scale (CARS) and the Social Responsiveness Scale (SRS). More significant improvements were observed in children under age 5 compared to those older.

A follow-up prospective study examined the effects of HBOT in 18 children with ASD [85]. Twelve children were treated at 1.3 atm/24% oxygen and 6 were treated at 1.5 atm/100% oxygen. Hyperbaric sessions were 45 minutes in duration for 40 total sessions. As previously noted, markers of oxidative stress and inflammation were measured. Pre- and post-HBOT parent-rated SRS and ATEC indicated significant improvements in each group, including motivation, speech, and cognitive awareness (p < 0.05 for each). Strengths of this study included the prospective nature and the use of objective measurements (oxidative stress and inflammatory markers). One group of investigators criticized this study, stating that significant improvements were only observed when both groups (1.3 atm and 1.5 atm) were combined [120]; however, the improvements were indeed significant for each group individually [85].

One small, prospective case series of 3 children with ASD using a multiple baseline design reported no significant improvements (compared to baseline) after 27–40 HBOT treatments at 1.3 atm/88% oxygen. The treatments were 1 hour in duration, 5 days per week. However, one child had an increase in spontaneous communication and another child had a decrease in problem behaviors with HBOT and an immediate increase in problem behaviors when HBOT was stopped [121]. These improvements were felt by the authors to be unrelated to HBOT but could not necessarily be explained by other factors. Strengths of this study included the prospective nature, the multiple baseline design (including a baseline prior to initiating HBOT), as well as evaluations by therapists and videotaping.

Another prospective study from Thailand reported the effects of HBOT at 1.3 atm/100% oxygen for 10 sessions (one session per week) in 7 children with ASD [122]. Significant improvements (p < 0.001 for each) were observed in social interaction, fine motor and eye-hand coordination, language, gross motor skills and self-help scores. Strengths of this study included the prospective nature and the objective measurements of self-help and motor skills by therapists.

A large, retrospective study from Turkey using HBOT at 1.5 atm/100% oxygen for 50 sessions at 60 minutes per day reported pre- and post-HBOT ATEC scores [59]. As previously noted, improvements in cerebral perfusion were observed on pre- and post-HBOT SPECT scans. As rated by clinicians/therapists for 54 children with ASD, improvements were observed in speech/language/communication in 79%, sociability in 85.5%, sensory/cognitive awareness in 87%, and health/physical/behavior in 75.2%. Strengths of this study included objective measurements (SPECT imaging), evaluations by clinicians, and a larger sample size than other studies.

One prospective study using a multiple baseline design examined the effects of HBOT at 1.3 atm/24% oxygen for 40 treatments in 16 children with ASD treated over an average of 56 days [123]. The mean frequency of treatments was 4.78 sessions per week with a range of 2.46 to 7.0 sessions. No consistent positive or negative effects were observed. The authors noted that the study used an observational technique which may not have been sufficient to measure changes in certain areas, such as attention and memory, and that the number of treatments per week was about half as other studies which reported improvements using similar HBOT parameters in children with ASD. Strengths of this study included the multiple baseline design (including a baseline prior to initiating HBOT), as well as evaluations by therapists and videotaping.

Finally, a more recent prospective study in 10 children with ASD measured the effects of HBOT at 1.5 atm/100% oxygen for 1 hour per day, 5 days per week for 80 treatments (completed over 20 weeks, with a 4 week break between the 40^th^ and 41^st^ treatment) on several behavioral scales as rated by parents and clinicians [86]. As previously noted, cytokine markers were measured before and after HBOT. Significant improvements were observed as measured by parent-rated ABC in irritability, lethargy, hyperactivity and overall scores (p = 0.02 or less for each). On the parent-rated PDD Behavior Inventory (PDD-BI), significant improvements were observed in sensory problems, specific fears, and aggressiveness (p = 0.006 or less for each). Overall, parents reported improvements in eye contact, imitation, language, tantrums, gastrointestinal problems and eczema. A significant improvement of 2 points (corresponding to “much improved”) was observed on the clinician-rated CGI-I scale in all 10 children. Strengths of this study included the prospective nature, evaluations by clinicians, and objective measurements (cytokine levels).

#### Studies with control children

In a recent systematic review published in *Medical Gas Research*, Ghanizadeh reviewed two randomized, double-blind, controlled trials using HBOT in children with autism [124]. The first study investigated the effects of HBOT at 1.3 atm/24% oxygen for 40 treatments, utilizing 2 treatments per day, 5 days per week, over 4 weeks in 33 children with autistic disorder compared to 29 control children with autistic disorder who received slightly pressurized room air (1.03 atm and 21% oxygen) [125]. Compared to the control group, significant improvements were observed in the treated children on the clinician-rated CGI scale and the parent-rated CGI and ATEC scales in outcomes including overall functioning, receptive language, social interaction, eye contact and sensory/cognitive awareness. Of the children completing more than 1 HBOT session, one child dropped out of the study after nine treatment sessions because asthma symptoms worsened, but this was not felt to be related to the treatment. Ghanizadeh noted that six other children dropped out of the study (four before the study began and two before finishing one full treatment). In this study, six medical centers participated and the findings did not significantly differ across centers. Strengths of this study included evaluations by blinded clinicians and parents (only the HBOT technician was aware of group assignment), an assessment of blinding (which was adequate), an intention-to-treat analysis (children finishing more than 1 HBOT session were included in the analysis), the prospective nature, the use of a control group, and the use of 6 centers (which may have minimized potential biases associated with a single site study).

Several criticisms of this study [125] arose in the comments sections of *BMC Pediatrics* and by other authors [120,123]. One criticism was the claim that the effect of treatment was determined by an intragroup analysis of the treatment group alone, and not by an intergroup analysis of the treatment compared to the control group; however, the analysis was indeed an intergroup analysis where the effects of treatment were compared between the two groups. The authors noted that a typographical error in the manuscript may have contributed to some confusion as ± SEM (standard error of the mean) was used when all of the reported values were actually ± SD (standard deviation). Another criticism was that the effect size of the treatment was probably small; however, the effect sizes were calculated as moderate to large (0.55 for the ATEC sensory/cognitive awareness subscale; 1.0 for physician-rated CGI score for overall functioning; and 0.62 for parent-rated CGI score for overall functioning [126]).

In the second controlled study, 18 children with autism were treated with HBOT at 1.3 atm/24% oxygen for 80 sessions (completed within 15 weeks) and compared to 16 children treated with placebo (free air-flow through a chamber at ambient pressure). Both groups received intensive ABA therapy and no significant changes were reported using several different behavioral scales [120]. Ghanizadeh [124] noted that twelve participants (26% of the children entering the study) withdrew from the study. It was not noted if these participants were in the treatment group or the control group or when they dropped out of the study; the scores from these 12 children were not included in the final analyses. Ghanizadeh [124] also observed that the number of patients in each group was small and that both groups showed some improvements during the study. Furthermore, it was noted by Ghanizadeh [124] that since both groups received intensive ABA therapy during the trial, one explanation for the lack of efficacy observed is that HBOT did not add significant therapeutic effects to intensive ABA. Strengths of this study included the prospective nature, the use of a control group and evaluation by blinded assessors.

Ghanizadeh [124] reported several important distinctions between these two controlled trials [120,125] which might help account for the different outcomes, including the number of participants, potential differences in diagnoses, different age ranges of the study participants, different outcome assessors, possible differences in demographics and autism severity, multicenter [125] vs. single center trial [120], assessment of blinding in one study [125] but not in the second [120], and the intensity of treatments with one study providing a mean of 10 hours of hyperbaric treatments per week [125] and the other study providing, on average, about 5 hours per week [120]. Ghanizadeh also noted that one [120] of the studies had a relatively high dropout rate which may have affected the results of the study. Although the other controlled study had 7 children drop out of the study, 4 dropped before starting the study and two before finishing one treatment session [125]. Moreover, Ghanizadeh noted that for one of the studies [120], there was no assessment of blinding efficacy as described in other HBOT studies [127,128].

### Adverse effects of HBOT in ASD

Most studies did not report any significant adverse events using HBOT in individuals with ASD. Some studies specifically noted there were no adverse events [85,119]. One study reported no adverse effects except for transient tinnitus in one child which resolved within one week [122]. Another study reported several non-serious adverse events, including ear discomfort (4 children), ear infections (2 children) and for 1 child each: hyperactivity, increased vocal sensitivity, increased sensory needs, insomnia, fatigue, dehydration, irritability, mouthing of objects, and a seizure [86]. One of the controlled studies reported that one child in the treatment group developed both urinary frequency (urinalysis was normal) and a skin rash that the treating physician thought was yeast-related. Another child in the treatment group had worsening of asthma symptoms after nine treatment sessions and was removed from the study; a third child had anxiety and dropped out of the study before finishing one full treatment. In the control group, one child developed abdominal distension and diarrhea during the study and another child had worsening of eczema [125]. The other controlled study reported no adverse events in the treatment group but reported that one of the children in the control group developed hyponatremia and the acute onset of seizures and was removed from the study [120]. In a recent systematic review, Ghanizadeh highlighted that the use of HBOT in children with ASD was associated with minimal adverse events.

### Limitations of the reviewed studies

Many of the reviewed studies suffered from limitations, including the lack of control children, an open-label design, a small number of participants, a retrospective design and the use of parent-rated scales. Indeed, there were only two controlled studies that did not suffer from these types of limitations. These limitations may have contributed to inconsistent findings across studies. In addition, some studies used measurements or observational techniques which may not have been sufficient to measure changes in certain areas, such as attention and memory [123]. The reviewed studies also utilized many different standardized and non-standardized instruments, making it difficult to directly compare the results of studies or to know if there are specific areas of behavior in which HBOT is most effective. None of the studies reported measurements of the long-term effects of HBOT beyond the study period, so it is not known if any of the reported improvements were long lasting.

Most of the reviewed studies relied on changes in behavioral measurements, which may lag behind physiological changes. Based on previous studies of ABA therapy in children with ASD, it is not likely that substantial changes in behavior will be detectable over short observation periods, i.e., less than one year. In fact, studies on ABA therapy report substantial changes over periods from 1 to 4 years [41-43]. Given the complex requirements of brain development, it is likely that the observed physiological and neuroimaging changes observed in children with ASD using HBOT precede developmental and intellectual improvements. In fact, the studies which examined physiological changes with HBOT, especially changes in cerebral perfusion, reported substantial changes which were often observed over short periods of time. Although many of the reviewed studies reported behavioral improvements in some children with ASD, none of the studies lasted more than several months. This time period is probably of insufficient length to quantify the impact of HBOT on development. Additional studies examining the long term effect of HBOT in individuals with ASD are warranted.

Most studies reported improvements with HBOT in physiological abnormalities and/or behavioral outcomes of children with ASD; however, two studies from the same group of researchers did not find any notable improvements [120,123], and a third small study reported minimal improvements [121]. The variability in results between studies could have been due to certain subgroups of children with ASD responding differently to HBOT [123]. For example, it is possible that children with abnormal cytokines, higher inflammatory markers, cerebral hypoperfusion or mitochondrial dysfunction may be more likely to demonstrate improvements. However, many of the behavioral studies did not measure changes in biochemical variables (such as markers of inflammation or oxidative stress). One behavioral study measured changes in cytokine levels, but all of the children treated with HBOT had normal cytokine levels, making it unlikely that a significant change in cytokines would be observed [86]. Additional studies enrolling children with ASD who have certain physiological abnormalities (such as inflammation or cerebral hypoperfusion) and which measure changes in these physiological parameters would be helpful in investigating this further.

Studies which used a higher frequency of HBOT sessions (e.g., 10 sessions per week as opposed to 5 sessions per week) generally reported more significant improvements. This appears to be consistent with studies in other neurological conditions such as traumatic brain injury [17] where studies using a higher mean number of HBOT sessions per month (e.g., 40 treatments within a one month period) generally reported more significant effects. Additional studies are needed to look at various HBOT parameters (pressure and oxygen levels) in children with ASD to help determine optimal treatment parameters.

## Conclusions

HBOT at the pressures commonly used in children with ASD (up to 1.5 atm/100% oxygen) has been reported to improve cerebral perfusion, decrease markers of inflammation and not worsen oxidative stress markers. Most studies of HBOT in children with ASD reported improvements in several behavioral domains although many of these studies were not controlled. Although the two trials employing a control group reported conflicting results, a recent systematic review noted several important distinctions between these trials. Collectively, the reviewed studies indicate that the use of HBOT in children with ASD is associated with minimal adverse events and is well tolerated. We conclude that HBOT is a safe and potentially effective treatment for children with ASD but that further studies are warranted. Future studies would be wise to use standardized behavioral measurement tools and physiological biomarkers in a controlled study design. Targeting ASD subgroups that possess specific physiological abnormalities with HBOT may be a potentially fruitful method for determining which ASD individuals would benefit from treatment with HBOT.

## Competing interests

DAR, CS, SHF, NO, SC, JAB and EAM treat individuals with hyperbaric treatment in their clinical practices and derive revenue from this. KVD works at a hyperbaric center and recommends HBOT, but does not derive revenue from hyperbaric treatments. JJB prescribes hyperbaric treatment but does not derive revenue from this. DAR and EAM have previously received research funding from the International Hyperbarics Association (IHA) for two studies of hyperbaric treatment in children with autism [85,125] and CS previously received research funding from the IHA for one of these studies [125]. JAB has previously received research funding from the IHA for one study of hyperbaric treatment in children with autism and their parents. EAM has received hyperbaric chambers and financial support from OxyHealth LLC for remodeling the Rimland Center, a center for mentoring clinicians interested in learning how to care for children with autism spectrum disorders. DAR and KVD are medical advisors (unpaid) for IHA. REF declares that he has no competing interests.

## Authors' contributions

DR conceived the study and wrote the initial draft. All remaining authors edited the paper for content and suggested changes. All authors read and approved the final manuscript.
